# Niemann–Pick Type C2 Proteins in *Aedes aegypti*: Molecular Modelling and Prediction of Their Structure–Function Relationships

**DOI:** 10.3390/ijms25031684

**Published:** 2024-01-30

**Authors:** Prathigna Jaishankar Thambi, Cassandra M. Modahl, R. Manjunatha Kini

**Affiliations:** 1 King’s College London, London SE1 1UL, UK; prathignathambi@gmail.com; 2 Department of Biological Sciences, Faculty of Science, National University of Singapore, Singapore 117543, Singapore; 3 Centre for Snakebite Research and Interventions, Liverpool School of Tropical Medicine, Liverpool L3 5QA, UK; 4 Vector Biology Department, Liverpool School of Tropical Medicine, Liverpool L3 5QA, UK; 5 Department of Pharmacology, Yong Loo Lin School of Medicine, National University of Singapore, Singapore 117600, Singapore; 6 Department of Biochemistry and Molecular Biology, VCU School of Medicine, Virginia Commonwealth University, Richmond, VA 23298, USA

**Keywords:** chemoattractant, vector-borne disease, 3D structure, binding residues

## Abstract

*Aedes aegypti* is a major vector that transmits arboviruses through the saliva injected into the host. Salivary proteins help in uninterrupted blood intake and enhance the transmission of pathogens. We studied Niemann–Pick Type C2 (NPC2) proteins, a superfamily of saliva proteins that play an important role in arbovirus infections. In vertebrates, a single conserved gene encodes for the NPC2 protein that functions in cholesterol trafficking. Arthropods, in contrast, have several genes that encode divergent NPC2 proteins. We compared the sequences of 20 *A. aegypti* NPC2 proteins to the cholesterol-binding residues of human and bovine, and fatty-acid-binding residues of ant NPC2 protein. We identified four mosquito NPC2 proteins as potential sterol-binding proteins. Two of these proteins (AAEL006854 and/or AAEL020314) may play a key role in ecdysteroid biosynthesis and moulting. We also identified one mosquito NPC2 protein as a potential fatty-acid-binding protein. Through molecular modelling, we predicted the structures of the potential sterol- and fatty-acid-binding proteins and compared them to the reference proteins.

## 1. Introduction

*Aedes aegypti* and *A. albopictus* are primary or secondary vectors of the dengue, chikungunya, and Zika viruses [1]. In gonotrophic cycles, female mosquitoes feed on host blood to obtain the nutrients necessary for producing viable eggs. During these blood meals, a ‘naïve’ mosquito acquires the pathogens circulating in the infected host blood. These pathogens initially reach the mosquito midgut, overcome physical and immune barriers, and multiply [2]. The presence of viral proteins and RNA can be seen in the midgut as early as 2–3 days after an infected blood meal, peaking around ten days [3]. The virus then disseminates to other regions of the mosquito’s body, eventually replicating in the salivary gland. Subsequently, the pathogens in the saliva are transferred to a healthy person during the next blood meal, thus spreading the virus.

During a bite, the mosquito injects its saliva into the host to regulate host responses to vessel injury such as blood coagulation, platelet aggregation, and vasoconstriction [4]. The infusion of saliva ensures continuous blood flow during the meal. Inadvertently, the saliva infusion aids in the transfer of viruses. Further, similar to saliva proteins from other haematophagous arthropods, mosquito saliva proteins enhance the infectivity of the pathogens [4]. Therefore, mosquito saliva proteins facilitate both blood intake and enhanced pathogen transmission. As a first step to understand their role, mosquito salivary proteins have been profiled using transcriptomes and proteomes [5,6,7,8,9,10]. These salivary proteins are classified into several superfamilies based on their similarity to various previously characterised proteins [11]. Each protein superfamily contains multiple isoforms encoded by distinct genes with varied sequences. Although these proteins share comparable disulphide pairings and three-dimensional structures, they may exhibit distinct biological functions. Only a small number of mosquito proteins have been characterised and their structure, function, and mechanism of action determined. The physiological functions of most of these proteins have yet to be established.

The members of one superfamily of mosquito saliva proteins share sequence similarity with the human Niemann–pick Type C2 (NPC2) protein, and are hence classified as the NPC2 protein family. Human NPC2 is a small protein found in the lysosome, belonging to the MD-2 lipid recognition domain family [12]. NPC2 and NPC1 (structurally unrelated to NPC2) are involved in the binding, trafficking, and distribution of free cholesterol out of lysosomes to other parts of the cell and contribute to cholesterol homeostasis in all cell types [12]. Mutations in either the NPC1 or the NPC2 protein can cause accumulation of cholesterol in lysosomal compartments and lead to a rare neurovisceral lipid storage disorder, Niemann–pick Disease Type C [12]. This rare autosomal recessive disorder affects 1 in 100,000 people and can be fatal [13].

A single highly conserved (chemically similar) gene encodes the NPC2 protein in mammals and other vertebrates, with an identity of 75% among mammals and 55–70% across vertebrates [14]. The high conservation of NPC2 proteins is probably associated with its single functionality. Unlike vertebrates, arthropods have several genes encoding structurally divergent NPC2 proteins. In *A. aegypti*, 13 NPC2 proteins are expressed in the salivary gland (Supplementary Table S1 in [8]). However, a genome-wide search indicates that there are 20 NPC2 genes (Genome version AaegL5.3) [15]. These NPC2 proteins probably play distinct biological roles. The structure–function relationships and the physiological roles of the NPC2 proteins are poorly understood. In this paper, we have compared *A. aegypti* NPC2 proteins to human and bovine NPC2 proteins that bind to cholesterol and ant NPC2 that binds to fatty acid. Based on the similarities, we have identified four and one NPC2 proteins that may bind to sterols and fatty acid, respectively. The three-dimensional structures of potential sterol and fatty-acid-binding proteins have been predicted through molecular modelling. These predicted protein structures and their binding residues were compared to those of reference proteins.

## 2. Results

### 2.1. Sequence Analysis

**Figure 1:**
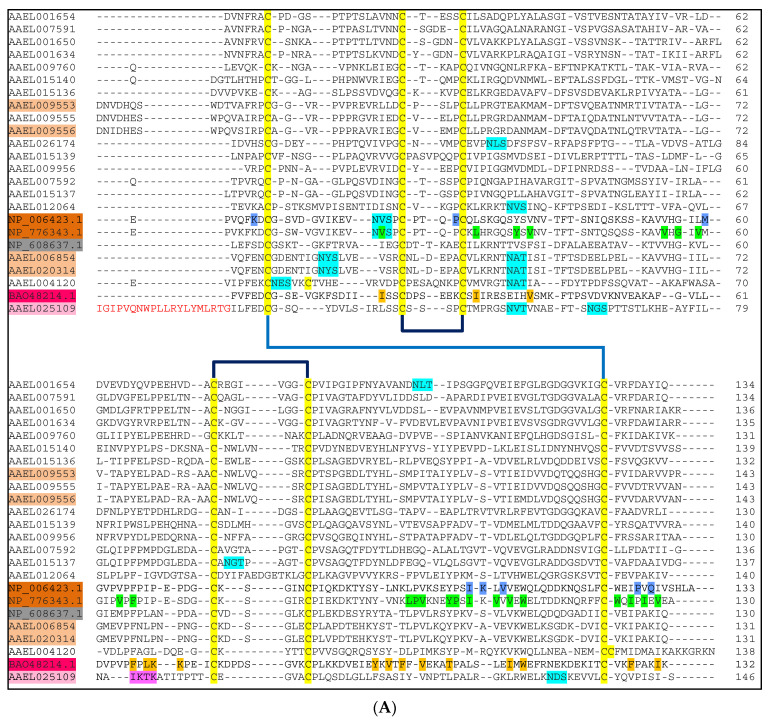

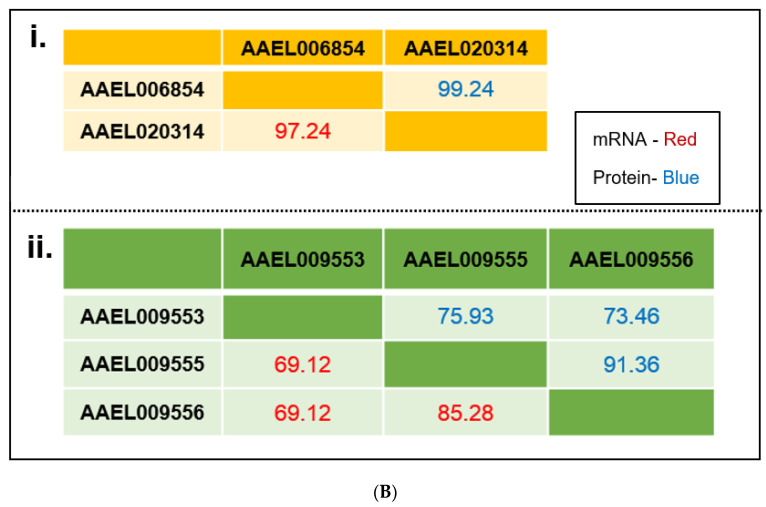
Sequence alignment of mature *Aedes aegypti* NPC2 proteins. (**A**) The mature NPC2 proteins of *Aedes aegypti* (AAEL0xxxxx)*, Homo sapiens* (NP_006423.1), *Bos taurus* (NP_776343.1)*, Camponotus japonicus* (BAO48214.1) and *Drosophila melanogaster* (NP_608637.1) were aligned using Clustal Omega. Six conserved cysteines are highlighted in yellow and the three predicted disulphide pairings (C1-C6, C2-C3, C4-C5) based on the crystal structures are shown. The predicted *N*-Glycosylation sites are highlighted in cyan. The residues involved in cholesterol binding in bovine NPC2 are highlighted in green, while the residues interacting with NPC1 and, hence, cholesterol transfer in human NPC2 are highlighted in blue. The accession numbers of cholesterol-binding bovine and human NPC2 are highlighted in dark brown, while those of predicted *A. aegypti* NPC2 proteins are highlighted in light brown. The *Drosophila* NPC2a is highlighted in grey. The residues involved in fatty acid binding in ant NPC2 are highlighted in orange. The accession numbers of fatty-acid-binding ant NPC2 and predicted *A. aegypti* NPC2 are highlighted in dark pink and light pink, respectively. The sequence pattern IKTK that functions as the double lysine site in AAEL025109 is highlighted in purple. (**B**) The mRNA and protein identity of closely related genes belonging to groups A (i) and B (ii) NPC2 genes are shown.

To identify whether the *A. aegypti* NPC2 proteins are secreted or cytosolic proteins, we analysed them for the presence of signal peptide. All *A. aegypti* NPC2 proteins have signal peptides at the N-terminal, and hence, they are most likely secreted proteins. We aligned mature amino acid sequences of *A. aegypti* NPC2 proteins with *Homo sapiens* (NP_006423.1), *Bos taurus* (NP_776343.1), *Camponotus japonicus* (BAO48214.1) and *Drosophila melanogaster* (NP_608637.1) NPC2 proteins (Figure 1). The mature *A. aegypti* NPC2 protein sequences ranged in length from 130 to 146 amino acid residues (Figure 1). AAEL026174 is the shortest, while AAEL025109 is the longest. Except for AAEL004120, which has eight cysteines, all the proteins have six conserved cysteines that form three disulphide linkages C1-C6, C2-C3, C4-C5 (Figure 1). Eight NPC2 proteins that have at least one *N*-glycosylation site are AAEL001654, AAEL0026174, AAEL015137, AAEL012064, AAEL006854, AAEL020314, AAEL004120, and AAEL025109 (Figure 1). Human and bovine NPC2 have one *N*-glycosylation site each, whereas ant NPC2 and the remaining 12 *A. aegypti* NPC2 proteins do not have any *N*-glycosylation sites (Figure 1). Only one *N*-glycosylation site is conserved in five *A. aegypti* NPC2 proteins (AAEL012064, AAEL006854, AAEL020314, AAEL004120, and AAEL025109).

The sequence identity of mature proteins ranged from 14 to 99% (Figures S1 and S2). The proteins with the highest identity AAEL006854 and AAEL020314 are 131 amino acid residues in length and differ only by one residue; alanine89 in AAEL006854 is replaced by valine89 in AAEL020314. AAEL009555 and AAEL009556 also show high identity of 91.36%. The nucleotide identity for this pair is 75.79%. AAEL025109 is the most distinct NPC2 protein, with only 14–25% identity to the others (Figure S2). AAEL007591 and AAEL025109 are the least identical proteins with 14% identity. Although human and bovine NPC2 share 80% identity, they share only 18–40% identity with *A. aegypti* NPC2 proteins, while the ant protein shares 14–37% identity with *A. aegypti* NPC2 proteins.

### 2.2. Genome-Wide Distribution

**Figure 2:**
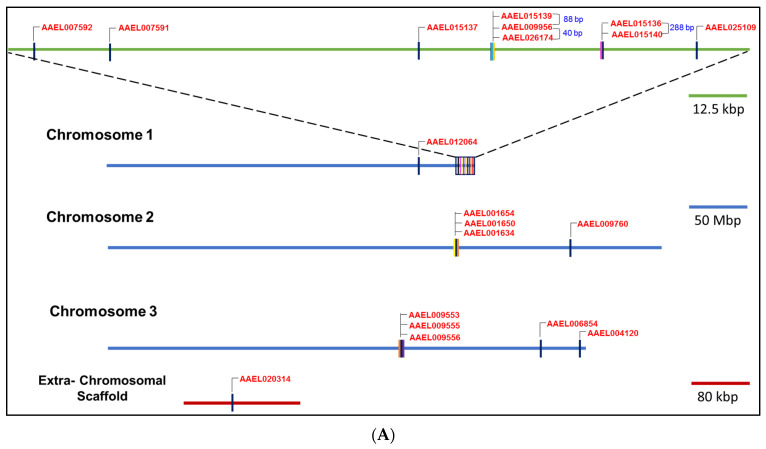

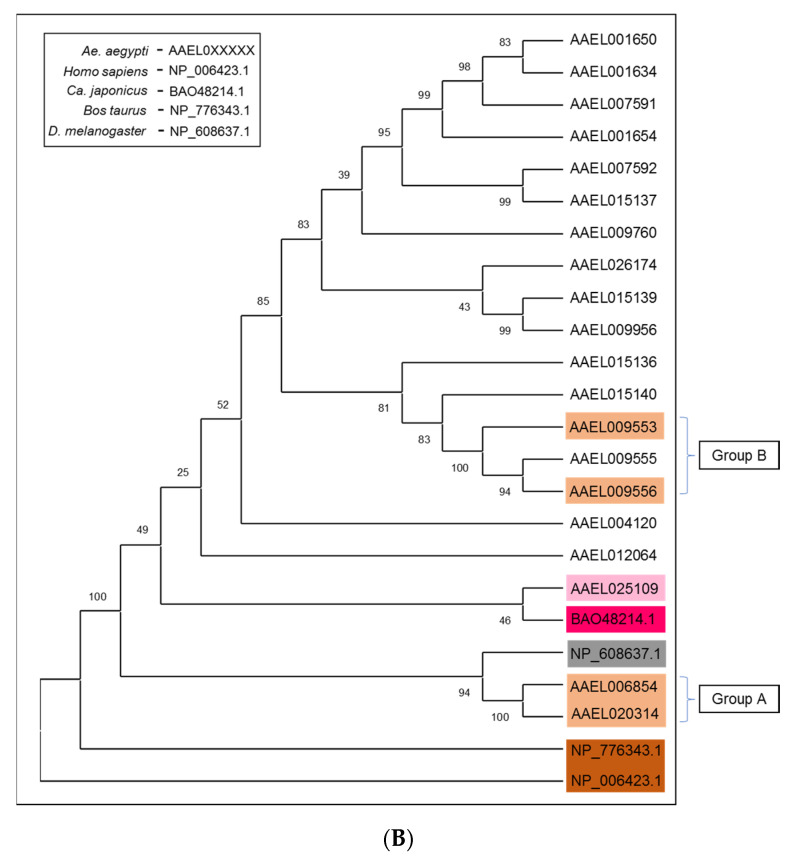
Genomic distribution and phylogenetic relationships of *A. aegypti* NPC2 genes. (**A**) Nineteen *A. aegypti* NPC2 genes are distributed among all three chromosomes, while one gene is located on an extra-chromosomal scaffold. The genome distribution is made to scale. Ten genes are located on chromosome 1, with nine genes forming a cluster. Within this cluster, two groups of three and two genes are separated by unusually small intergenic segments (indicated in bp). Four genes are located on chromosome 2, with three genes forming a cluster. Five genes are distributed on chromosome 3, with three genes forming a cluster. (**B**) Phylogenetic tree of *A. aegypti* NPC2 proteins along with bovine (2HKA), human (5KWY), and ant (3WEB) NPC2 proteins was constructed using MUSCLE and Mega X. This tree shows the diversity of the mosquito NPC2. The cholesterol-binding bovine and human NPC2 proteins are highlighted in dark brown, while those of predicted *A. aegypti* NPC2 proteins are highlighted in light brown. The *Drosophila* NPC2a is highlighted in grey. The accession numbers of fatty-acid-binding ant NPC2 and predicted *A. aegypti* NPC2 are highlighted in dark pink and light pink, respectively. Groups A and B NPC2 proteins have been identified.

NPC2 genes are distributed among all three chromosomes of *A. aegypti*, except for the AAEL020314 gene, which is located on an extra-chromosomal scaffold (Figure 2A). Ten genes are located on chromosome 1. Interestingly, nine of them form a cluster and lie within the 1.6 Mbp segment—AAEL007592, AAEL007591, AAEL015137, AAEL015139, AAEL009956, AAEL026174, AAEL015136, AAEL015140, and AAEL025109. Remarkably, AAEL009956 and AAEL026174 are the closest with an intergenic distance of only 40 bp (Figure 2A), followed by AAEL015139 and AAEL009956 (separated by 88 bp), and AAEL015136 and AAEL015140 (separated by 288 bp). In all these cases, the promoter regions of AAEL026174, AAEL015139, and AAEL015136 appear to be unusually short. Interestingly, despite their proximity, these genes share only low identity between their mRNA (35–53%) and protein (13–47%) sequences with the exception of AAEL007592 and AAEL015137, which share over 73% identity (Figure S1). The last gene, AAEL025109, is unusually close to the telomere, with only 7.7 Mbp from the end of the chromosome. Four genes are found on chromosome 2, where three genes are clustered together—AAEL001654, AAEL001650, and AAEL001634 (Figure 2A). Even though these genes are in a cluster, they only share about 58–72% mRNA identity and 49–69% protein identity. Five genes are found on chromosome 3, including one cluster of three genes—AAEL009553, AAEL009555, and AAEL009556 (Figure 2A). Two genes, AAEL009555 and AAEL009556, are 280 kbp apart and have 85.28% and 91.36% mRNA and protein identity, respectively (Figure 1). AAEL009553 shares 69.12% and 69.12% mRNA and 75.93% and 73.46% protein identity with AAEL009555 and AAEL009556, respectively (Figure 1).

### 2.3. Intron–Exon Structures of NPC2 Genes

We analysed intron–exon structures, chromosome location, gene and protein sizes, and the direction of transcription of all NPC2 genes (Figure S3). Most genes are less than 1200 bp long, ranging from 465 to 1135 bp. The AAEL012064, AAEL020314, AAEL006854, AAEL025109, and AAEL009760 genes, ranging from 14,000 to 41,500 bp, are the exceptions (Figure S3). The shortest and the longest genes with lengths of 465 bp and 41,500 bp are AAEL015137 and AAEL012064, respectively. The intron–exon structures are also varied. Fourteen genes contain only one intron and two exons (Figure S3). Only one gene (AAEL004120) has two introns and three exons, and three genes (AAEL025109, AAEL006854 and AAEL020314) contain three introns and four exons (Figure S3). AAEL012064 has the highest number of introns (five), two of which are in the 5′ UTR. Only AAEL015137 has no introns. Since it does not contain a poly (A) tail, it is most likely not a retrogene. Interestingly, AAEL020314 (extrachromosomal) and AAEL006854 share high protein identity (99.24%) but do not share the synteny. There was no matching nucleotide sequence identity in the vicinity (15 kbp on either side) of these two genes.

### 2.4. Structure–Function Relationships of NPC2 Proteins

#### 2.4.1. NPC2 Proteins in Cholesterol Binding and Transport

As mentioned in the Introduction, the mammalian NPC2 proteins play a pivotal role in cholesterol binding and egress of the lipid from the lysosome to other organelles [12]. The bovine NPC2 (PDB 2HKA) protein has an immunoglobulin-like fold stabilised by three disulphide bridges [16]. Apo NPC2 (PDB 1NEP) has multiple small loosely packed hydrophobic cavities which are closed. When cholesterol is bound, a deep hydrophobic cavity flanked by two β strands expands by repositioning the side chains of several residues to fit the ligand [16]. This malleability is a characteristic of vertebrate NPC2 proteins, unlike many lipid-binding proteins, which have large pre-existing cavities to bind various ligands [16].

**Figure 3:**
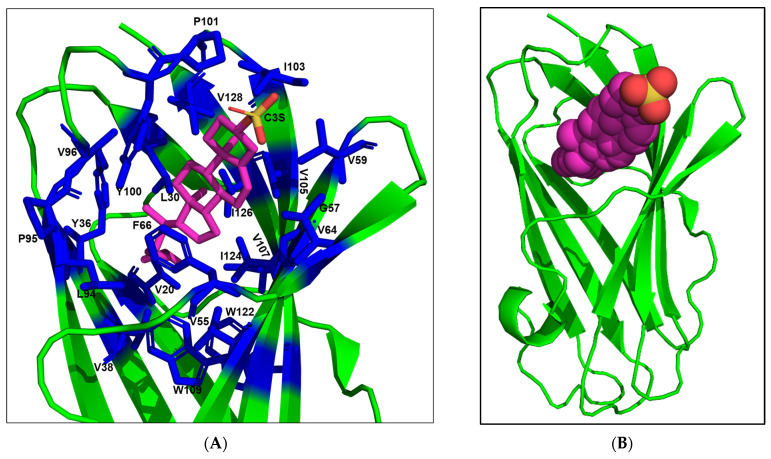

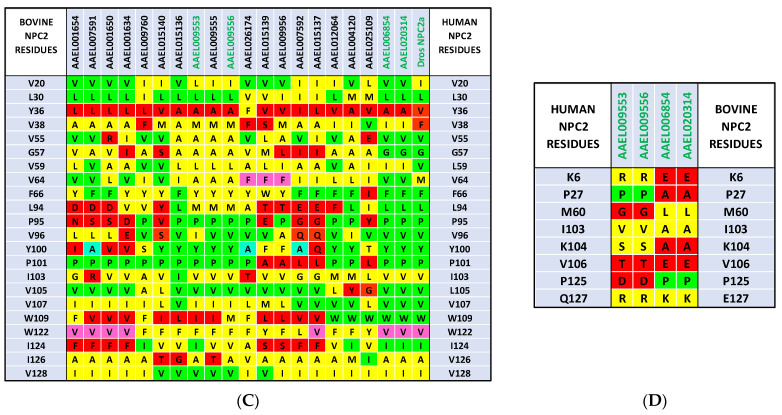
Cholesterol-binding site in NPC2 proteins. (**A**) Ribbon representation of bovine NPC2 bound to cholesterol sulphate (2HKA) is shown using PyMOL [19]. Cholesterol sulphate is depicted as a CPK model. Carbon, magenta; oxygen, red; and sulphur, yellow. (**B**) The residues involved in forming the predominantly hydrophobic tunnel are depicted as stick model (blue). (**C**) Comparison of amino acid residues of *A. aegypti* and *Drosophila melanogaster* NPC2a protein with those in cholesterol-binding site of bovine NPC2. Identical residues are highlighted in green, conserved residues in yellow, and non-conserved residues in red. Mutations studies indicated that the residues highlighted in teal show severe reduction, while those in violet have little to no impact on cholesterol binding. (**D**) Comparison of amino acid residues of four *A. aegypti* potential cholesterol-binding NPC2 proteins with those in human NPC2 involved in interaction with NPC1. Identical residues are highlighted in green, conserved residues in yellow, and non-conserved residues in red.

The cholesterol-3-*O*-sulphate molecule is bound to a predominantly hydrophobic sterol-binding tunnel of bovine NPC2 formed by 22 amino acid residues, V20, L30, Y36, V38, V55, G57, V59, V64, F66, L94, P95, V96, Y100, P101, I103, V105, V107, W109, W122, I124, I126, and V128 [16] (Figure 3A,B). The side chains of ten residues reposition when cholesterol is bound, L30, Y36, V38, F66, L94, P95, V96, Y100, P101, and I103 [16]. The entrance of this tunnel is formed by six residues—V59, V64, F66, Y100, P101 and I103 [16]. Cholesterol sulphate interacts with all these residues. Particularly, the two aromatic F66 and Y100 residues are involved in most interactions. The mutation of these aromatic residues to aliphatic Alanine residue severely compromises cholesterol binding [17]. Thus, their aromaticity is thought to contribute to protein structural stability as well as to strong hydrophobic interactions with cholesterol. Similarly, the replacement of V96, located in the middle of the cavity, with Phenylalanine hinders cholesterol binding due to steric clash [17]. On the other hand, V64F and W122A mutations have little impact on ligand binding [17]. P101 and V20, situated at the top and the base of the hydrophobic tunnel (Figure 3B), are crucial residues that form hydrophobic interactions with cholesterol. In Niemann–Pick disorder patients, P101S and V20M mutations lead to a loss of function [18].

All mosquito NPC2 proteins were analysed for the 22 amino acid residues involved in cholesterol binding. In Figure 3C, we highlighted all identical residues in green, conserved residues in yellow, and non-conserved (chemically dissimilar) residues in red. In all *A. aegypti* NPC2 proteins, the aliphatic residues V20, L30, V59, V107 and V128 are either conserved or replaced with other aliphatic amino acid residues (Figure 3C). The Y100A mutation, which results in loss of cholesterol binding [17], is found in AAEL007591, AAEL007592, and AAEL026174 (Figure 3C). Thus, these three proteins probably do not bind cholesterol or related molecules. The mutations V20M, F66A, and V96F that also result in a loss of cholesterol binding [18] are not found in *A. aegypti* NPC2 proteins. We speculate that the F66I mutation found in AAEL025109 (Figure 3C), similar to F66A, may lead to a loss of cholesterol binding. Twelve proteins, AAEL001654, AAEL007591, AAEL001650, AAEL001634, AAEL009760, AAEL015139, AAEL009956, AAEL007592, AAEL015137, AAEL015140, AAEL009555 and AAEL025109, have one or more polar substitutions in the hydrophobic tunnel, making it unsuitable for cholesterol binding (Figure 3C). Further, the replacement of P95 or P101 in these proteins can substantially compromise their conformation. Hence, these 12 proteins are probably not involved in cholesterol binding. On the other hand, the mutation V64F that had little impact on cholesterol binding [17] was found in AAEL026174, AAEL015139, and AAEL009956 (Figure 3C). In AAEL009556, the cholesterol-binding pocket is mostly intact except for a single mutation, Y36A (Figure 3C). AAEL006854 and AAEL020314, on the other hand, have two mutations: Y36A and W122V. As W122A showed to have little impact on cholesterol binding [17], we assume that the W122V mutation may also have little impact due to the aliphatic, hydrophobic nature of Alanine and Valine. AAEL009553 contains two mutations, Y36A and W109I (Figure 3C). We speculate that the mutation of W109 to an aliphatic, hydrophobic residue in the hydrophobic tunnel may have a limited effect on cholesterol binding. Based on the above analysis, we classify four proteins, AAEL009556, AAEL006854, AAEL020314, and AAEL009553, as potential cholesterol-binding proteins. Interestingly, two of these proteins, AAEL006854 and AAEL020314, show 51.16% identity with the *Drosophila* NPC2a (NP_608637.1) (Figure 1). Further, NPC2a retains most of the cholesterol-binding residues of bovine NPC2 (Figure 3C). In in vivo experiments, *Drosophila* NPC2a mutants show disordered cholesterol trafficking, leading to the accumulation of cholesterol [12]. Since *Drosophila* NPC2a plays an important role in sterol homeostasis and ecdysteroid biosynthesis [12], we hypothesise that AAEL006854 and/or AAEL020314 may play key role in ecdysteroid biosynthesis and, hence, moulting. Another two potential cholesterol-binding proteins, AAEL009553 and AAEL009556, may be involved in other physiologic function(s) by binding to sterols.

After binding to cholesterol, NPC2 interacts with NPC1 to form a cholesterol transfer complex [16]. Following this transfer, NPC1 transports cholesterol to other cell organelles to maintain the cholesterol homeostasis [16]. The crystal structure of the human NPC1-NPC2 complex (PDB 5KWY) reveals the residues involved in the interaction between NPC2 and the middle, luminally oriented domain (MLD) of NPC1. NPC1-MLD binds at the top of the cholesterol-binding tunnel of NPC2 primarily through its two projecting loops [20]. The binding interface consists of residues that are involved in both hydrophobic and hydrophilic interactions. Eight NPC2 residues, K6, P27, M60, I103, K104, V106, P125, and Q127, form the binding site [20]. Four of these eight residues, K6, M60, K104, and Q127, have distinct orientations in apo and cholesterol bound structures. Interestingly, M60 is involved in hydrophobic and hydrophilic interaction with NPC1 [20]. We analysed all four potential cholesterol-binding proteins from *A. aegypti* for these eight residues. Seven out of eight (87.5%) residues are mutated in each NPC2 protein; 50% of these mutations are conserved, while the other 50% mutations are non-conserved (Figure 3D). The key residue M60 is replaced by residues Gly/Leu in all the proteins (Figure 3D). Aliphatic V106 is replaced by a polar (charged Glu or uncharged Thr) residue, resulting in decreased hydrophobicity. P27A mutation is found in AAEL006854 and AAEL020314, whereas P125D mutation is found in AAEL009553 and AAEL009556 (Figure 3D). As unfavourable mutations are observed in all the potential cholesterol-binding NPC2 proteins, we do not expect *A. aegypti* NPC2 proteins to interact with human NPC1-like proteins. Instead, they may interact with *A. aegypti* NPC1 proteins (AAEL009531 and AAEL019883). Both these proteins have non-conserved mutations (Figure S4) replacing all the six residues of human NPC1 (Q421, Y423, P424, F503, F504, Y506) that interact with NPC2 [20]. Due to such drastic changes in the interaction sites in both *A. aegypti* NPC1 and NPC2 proteins, we cannot speculate whether any of *A. aegypti* NPC2 proteins would form complex with *A. aegypti* NPC1 proteins and are involved in cholesterol transfer or not. It may be possible that *A. aegypti* NPC2 proteins bind to sterols to perform other physiological functions. Interestingly, the pair NPC1 (AAEL009531) and NPC2 (AAEL001650) is suggested to function together and negatively affect the immune deficiency immune signalling pathway in mosquitos infected with dengue virus serotype 2 [21].

#### 2.4.2. Molecular Modelling of Potential Cholesterol-Binding NPC2 Proteins

**Figure 4:**
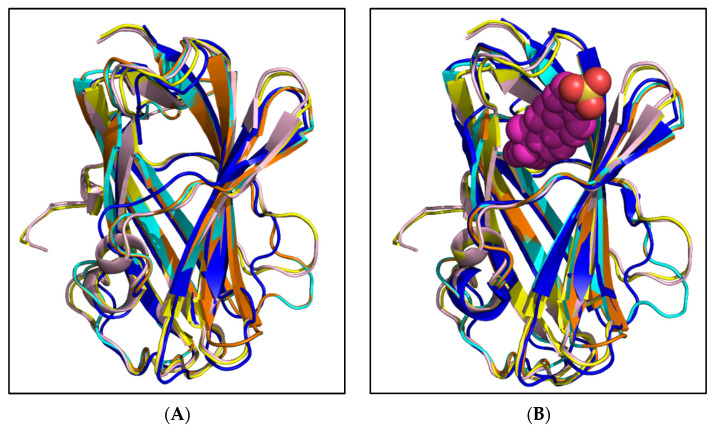
Modelled structures of potential cholesterol-binding mosquito NPC2 superimposed on apo and cholesterol-bound bovine NPC2. Ribbon representation of predicted structures of four potential cholesterol-binding mosquito NPC2 proteins, AAEL006854 (teal), AAEL020314 (orange), AAEL009553 (yellow), and AAEL009556 (pink) superimposed on apo (**A**) and bound (**B**) bovine NPC2 protein, 1NEP (red) and 2HKA (blue), respectively. The cholesterol sulphate is depicted in a CPK model.

**Figure 5:**
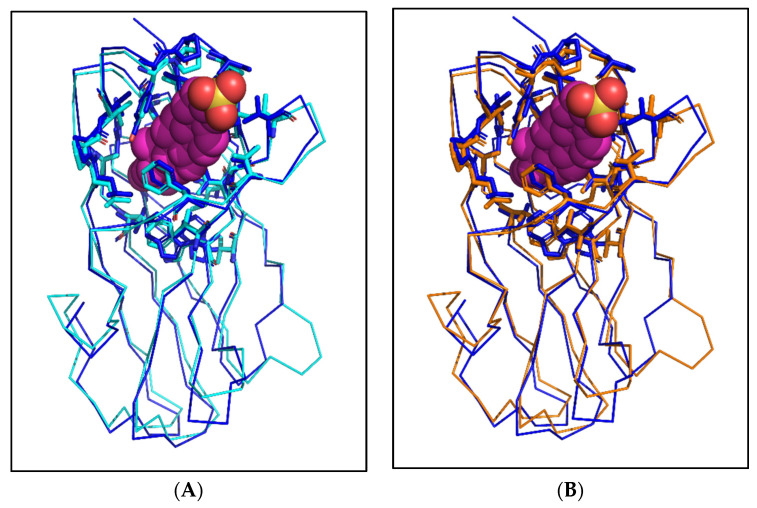

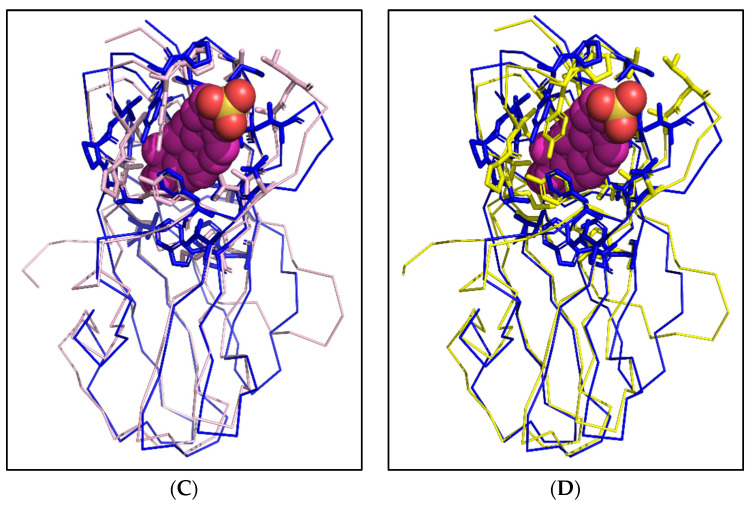
Cholesterol-binding residues of mosquito NPC2 proteins. Comparison of the binding residues of each reference mosquito NPC2 proteins, (**A**) AAEL006854 (teal), (**B**) AAEL020314 (orange), (**C**) AAEL009556 (pink) and (**D**) AAEL009553 (yellow), with that of bovine NPC2 (blue). The cholesterol sulphate is depicted as a CPK model.

The structures of the potential cholesterol-binding proteins, AAEL006854, AAEL020314, AAEL009553, and AAEL009556, were predicted using AlphaFold2. The pLDDT and PAE graphs were analysed (Figure S5A,B). The top rank, low PAE and high pLDDT structures were concluded to be the best models for each protein. Further, each of the proteins were compared to apo and bovine NPC2 structure on PyMOL [19]. Superimposition of each of the predicted structure using the function ‘super’ helped align the structures with apo and bound bovine NPC2 (Figure 4A,B). As expected, the structures of mosquito NPC2 aligned remarkably with apo and bound bovine NPC2 with RMSD ranging between 0.7 and 1.2 Å (Table S1). The alpha helices, beta sheets, and loops of most parts are aligned well. The most variable regions are the loops at the N terminal end of each NPC2. We predict that this could be due to deletion and the varied positions of cysteine and proline in the sequences. The binding residues of potential cholesterol-binding mosquito NPC2 proteins are in similar positions to that of bovine NPC2 (Figure 5A–D). As discussed above, the slight changes in a few residues still create acceptable space for the NPC2 binding pocket to fit cholesterol.

#### 2.4.3. NPC2 Proteins in Semiochemical Binding and Communication

**Figure 6:**
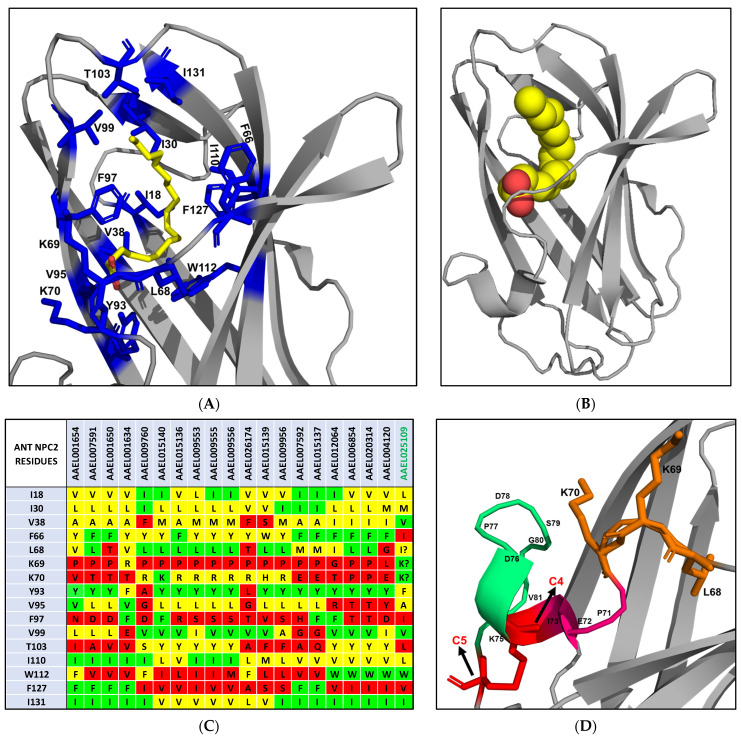
Fatty-acid-binding site in NPC2 proteins. (**A**) Ribbon representation of ant NPC2 bound to oleic acid (3WEB) is shown using PyMOL [19]. Oleic acid is depicted as a CPK model. Carbon, yellow; and oxygen, red. (**B**) The residues involved in forming the cavity are depicted as stick model (blue). (**C**) Comparison of amino acid residues of *A. aegypti* NPC2 proteins with those in fatty-acid-binding site of ant NPC2. Identical residues are highlighted in green, conserved residues in yellow, and non-conserved residues in red. (**D**) The segment of double lysine site that is in a loop in the ant protein. In AAEL025109, the absence of series of prolines allows the β strand to extend. The two lysine residues would then be on the same surface of the β strand available to bind oleic acid.

NPC2 proteins in arthropods help in the recognising and binding of semiochemicals, thereby regulating the chemosensory activity [22,23]. The NPC2 protein of ant *Camponotus japonicus* is expressed in the antenna and it binds to various semiochemicals, such as long-chain fatty acids and alcohols [22]. Similar to the mammalian NPC2, ant NPC2 has a β-sandwich fold. However, it has a large ligand-binding cavity in the interior [22]. The crystal structure of the ant NPC2–oleic acid complex (PDB 3WEB) reveals that the ligand is bent into a U-shaped conformation to fit into the binding pocket (Figure 6A). Interestingly, the polar carboxylic acid group of oleic acid is facing the bottom of the hydrophobic cavity, while the polar sulphate group of cholesterol sulphate is fully exposed at the top of the hydrophobic cavity (Figure 3A and Figure 6A). The binding pocket of ant NPC2 is formed by 16 amino acid residues, namely I18, I30, V38, F66, L68, K69, K70, Y93, V95, F97, V99, T103, I110, W112, F127, and I131 (Figure 6B). To better accommodate the ligand, W112 has distinct orientations in the apo and bound structures, i.e., it flips its side chain by 120° to avoid a steric clash with oleic acid. Ant NPC2 has a distinct double lysine site, K69 and K70, that forms two hydrogen bonds with O1 and O2 atoms of oleic acid [22]. These bonds are crucial for the high-affinity binding of oleic acid [22]. K69P and K70N/Q mutations are commonly found in other arthropods and vertebrates. This significant change explains the low affinity of fatty acids to mammalian NPC2 [22].

All mosquito NPC2 proteins were analysed for the 16 amino acid residues involved in fatty acid binding. In all the proteins, three residues—I18, I30, and I131—are either conserved or replaced by conserved aliphatic amino acid residues (Figure 6C). W112, a residue that changes its orientation as mentioned above, is conserved in AAEL012064, AAEL006854, AAEL020314, AAEL004120, and AAEL025109 (Figure 6C), while it is replaced by F112 in AAEL001654, AAEL009760, and AAEL026174. In the rest of the proteins, W112 is replaced by an aliphatic amino acid residue (Figure 6C). The double lysine site that is critical for oleic acid binding is mutated in most *A. aegpyti* NPC2 proteins. The K69P mutant is found in 16 proteins, making it unfavourable for hydrogen bonding (Figure 6C). AAEL012064 and AAEL004120 have K69G and K69L mutations, respectively. K69R mutation is found in AAEL001634 (Figure 6C). On the other hand, K70 is mutated in 18 proteins. Seven proteins have a conserved mutation of K70R and one protein has a mutation of K70H (Figure 6C). The rest of the proteins have non-conserved mutations. K70 residue is conserved in AAEL015140 and AAEL025109. We speculate that the mutation of K69 or K70 to a polar, basic residue (Arg/His) may allow the protein to form a single hydrogen bond with oleic acid, although the binding affinity could be significantly affected. F97, which forms three hydrophobic interactions with the ligand, is replaced with a polar charged/uncharged or aliphatic amino acid residue in most of the proteins, resulting in a reduction in hydrophobicity. Hence, we speculate that 19 of 20 mosquito NPC2 proteins may not be involved in fatty acid binding. One protein, AAEL025109, contains four mutations, F66I, F97I, T103L and F127V (Figure 6C). The two conserved K69 and K70 residues that are in a loop of ant NPC2 (Figure 6D) are replaced with IKTK in a β-strand. The absence of a series of proline residues (PVPFPLKKPE) in the vicinity allows the 6th β strand to extend, subsequently allowing these two lysine residues to potentially be available on the same surface of the β strand for hydrogen bonding with oleic acid. Based on this analysis, we conclude that AAEL025109 may be a potential fatty-acid-binding protein.

#### 2.4.4. Molecular Modelling of Potential Fatty-Acid-Binding NPC2 Protein

**Figure 7:**
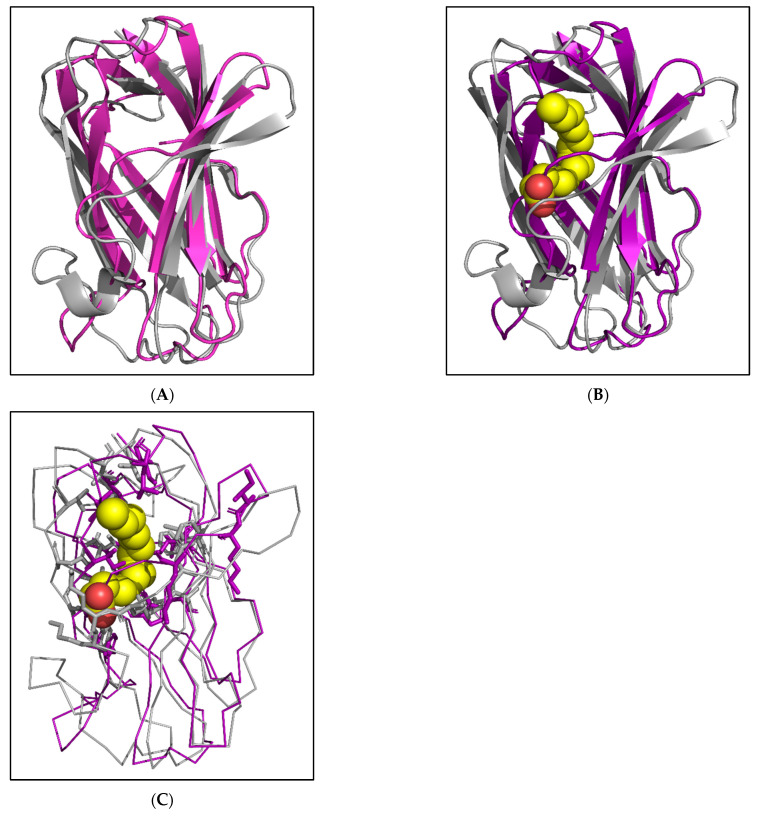
Modelled structure of potential fatty-acid-binding mosquito NPC2 protein. Ribbon representation of predicted structure of potential fatty-acid-binding mosquito NPC2 protein, AAEL025109 (purple) superimposed on apo (**A**) and bound (**B**) bovine NPC2 protein, 3WEA (brown) and 3WEB (ash), respectively. The oleic acid is depicted as a CPK model. (**C**) Comparison of the binding residues of reference mosquito NPC2 protein with that of ant NPC2.

The N terminal end of the potential fatty-acid-binding protein, AAEL025109, is unusually long with 20 additional residues (highlighted in red in Figure 1). Therefore, this part of the protein was removed before molecular modelling to aid in correct structure prediction. The structure of the potential fatty-acid-binding protein AAEL025109 was predicted using AlphaFold2. The top rank, low PAE and high pLDDT structure was concluded to be the best model (Figure S6A,B). Further, the protein was compared to apo and bound ant NPC2 structure on PyMOL through superimposition (Figure 7A,B). The structure of mosquito NPC2 aligned well with apo and bound ant NPC2 with RMSD 2.0 Å and 2.4 Å, respectively (Table S2). The binding residues of potential fatty-acid-binding mosquito NPC2 protein are in similar positions to that of ant NPC2 (Figure 7C). As predicted, the absence of proline residues allows the 6th β strand to extend. However, because of the additional residues, these lysines are located far away from the K69 and K70 of ant NPC2. Thus, oleic acid (or fatty acid or long-chain alcohol) may bind to AAEL025109 in a different orientation where K69 and K70 are available for strong hydrogen binding. Additionally, this could be due to misalignment of K69 and K70 as there are four Pro residues in BAO48214.1 (all disruptors of secondary structure) compared to one Pro residue in AAEL025109, making it difficult to determine the correct alignment.

### 2.5. Expression of Potential Cholesterol- and Fatty-Acid-Binding NPC2 Genes

The potential cholesterol-binding NPC2 genes have a varied expression in the tissues of adult female *A. aegypti*. While AAEL006854 and AAEL020314 are expressed highly in the antenna, brain, and proboscis, AAEL009553 and AAEL009556 are expressed in small amounts only in the antenna and brain, respectively (Table S3A). AAEL006854 and AAEL009556 are expressed in the midgut and salivary gland, whereas AAEL020314 and AAEL009553 are expressed only in the midgut and salivary gland, respectively (Table S3B,C). The varied expression of the potential cholesterol-binding genes in different mosquito tissues may indicate different roles played by them in these tissues.

The potential fatty-acid-binding gene, AAEL025109, is not expressed in detectable levels in adult female *A. aegypti* tissues of the antenna, brain, proboscis, salivary gland and midgut (Table S3A–C). It is unclear whether it is expressed in other tissues, in male mosquitos or in other developmental stages of mosquito life cycle.

### 2.6. Phylogenetic Analysis

Phylogenetic analysis was conducted on mature protein sequences (Figure 2B). The potential cholesterol-binding proteins are highlighted in rust and the potential fatty-acid-binding proteins in lavender (Figure 2B). Potential cholesterol-binding AAEL006854 and AAEL020314 are evolutionarily distinct from all other *A. aegypti* NPC2 proteins, including the other two putative cholesterol-binding AAEL009553 and AAEL009556. As expected, AAEL006854 and AAEL020314 are clustered with *Drosophila* NPC2a (NP_608637.1). Although still closely related to other *A. aegypti* NPC2 proteins, the putative fatty-acid-binding AAEL025109 is clustered with the ant fatty-acid-binding NPC2 (Figure 2B).

### 2.7. Expression of NPC2 Genes Post Arbovirus Infection

Gene expressions in the midgut (infection initiation site—midgut infection barrier) and salivary gland (pathogen transmission site—salivary gland infection barrier) after an arbovirus infection have been documented [6,8,9,24]. We analysed the differential expression of NPC2 genes in uninfected and infected mosquito midgut and salivary gland tissues to understand the potential role of NPC2 proteins during infection. Transcriptome data from the midgut (1-day post infection (dpi), 4-dpi and 14-dpi for CHIKV, DENV1 or DENV2 with suitable controls, respectively) and salivary gland (7-dpi for CHIKV and 14-dpi for DENV2 or ZIKV) were examined [6,8,9,24]. We did not analyse the gene expression data of the brain, antenna and proboscis after an infection, as the roles of these tissues in virus infection have not yet been established.

#### 2.7.1. Differential Expression in the Midgut

Eleven NPC2 genes showed differential expression in the midgut of uninfected and CHIKV-infected mosquitos [9]. Six genes, AAEL001650, AAEL001654, AAEL006854, AAEL012064, AAEL004120 and AAEL009556, are downregulated. While the expressions of AAEL001650, AAEL001654 and AAEL006854 are turned down to zero, those of AAEL012064, AAEL004120 and AAEL009556 are downregulated by 1.9-, 1.7- and 1.4-fold, respectively (Table S4A and S4B). On the other hand, five genes, AAEL009555, AAEL020314, AAEL007591, AAEL015137 and AAEL009760, are upregulated. AAEL009555 is only expressed post infection. AAEL020314 is upregulated by 2.0-fold, and AAEL007591, AAEL009760 and AAEL015137 are each upregulated by about 1.5-fold (Table S4A,B).

We analysed the differential expression of NPC2 genes in uninfected and infected mosquitos, which had been infected with two different serotypes of DENV—DENV1 and DENV2 [9,23]. In the case of DENV1 at 4-dpi in the Thailand mosquito strain, 14 genes are downregulated. The expression of AAEL001650, AAEL001654 and AAEL009956 is fully turned off post DENV1 infection (Table S4C,D). The order of differential expression for eleven genes along with their fold change is AAEL007591 (76.3×) > AAEL015137 (75.7×) > AAEL015136 (54.7×) > AAEL015140 (24.5×) > AAEL009556 (22.4×) > AAEL006854 (14.3×) > AAEL004120 (10.7×) > AAEL026174 (9×) > AAEL020314 (4.6×) > AAEL012064 (4×) > AAEL007592 (1.5×) (Table S4C,D). On the other hand, AAEL009760 (14.6×) is the only gene that is upregulated post DENV1 infection (Table S4D).

In the case of DENV2 at 14-dpi in the Chetumal mosquito strain, five genes are downregulated [6]. The expression of AAEL009555 is turned off completely post DENV2 infection (Table S4E). The order of differential expression for four genes along with their fold change is AAEL020314 (7×) > AAEL012064 (2.4×) > AAEL004120 (1.9×) > AAEL009556 (1.2×) (Table S4E,F). On the other hand, four genes are upregulated in the following order: AAEL015140 (2.8×) > AAEL001650 (1.8×) > AAEL006854 (1.6×) > AAEL009760 (1.2×) (Table S4E,F). Independent studies also show the upregulation (2.2×) of AAEL001650, and AAEL006854 was observed 7-dpi in the DENV2-infected midgut of Rockefeller (laboratory-maintained) and Caribbean (field-derived) strains of mosquitos [21]. Although both genes were silenced in these strains of mosquitos, only AAEL001650 showed a significant decrease in median DENV2 titre in the midgut, while the silencing of AAEL006854 had no significant effect on infection [21]. In a recent study, the knockdown of gene AAEL015136 lead to a decrease in DENV2 infection in the Cali-S susceptible strains, whereas it increased the infection rate in the Cali-MIB refractory strains [24]. The authors concluded that such a contradictory observation is probably due to the distinct roles played by AAEL015136 in different mosquito strains [24]. This gene did not show any significant differential expression in the Chetumal strain of mosquitos (Table S4E).

In summary, the NPC2 genes AAEL004120, AAEL012064 and AAEL009556 are downregulated post all three (CHIKV, DENV1 and DENV2) infections, whereas AAEL009760 is upregulated post all three infections. Four genes, AAEL004120, AAEL020314, AAEL012064 and AAEL009556, are downregulated in both DENV1 and DENV2, whereas AAEL001650 and AAEL006854 are both downregulated in DENV1 and CHIKV. Interestingly, AAEL001650, AAEL015140 and AAEL006854 showed contrasting results in midgut infected with DENV1 and DENV2. They are downregulated in DENV1 and upregulated in DENV2. These observed differential expression profiles of NPC2 genes may be due to either viral serotypes (DENV1 vs. DENV2), distinct mosquito strains (Thailand vs. Chetumal) or even different time points post infection (4-dpi vs. 14-dpi). Overall, the above analysis on the differential expression in the midgut showed that NPC2 genes are more downregulated than upregulated in midgut infected with CHIKV, DENV1 or DENV2.

#### 2.7.2. Differential Expression in the Salivary Gland

In CHIKV-infected salivary gland at 7-dpi, six genes, AAEL012064, AAEL001634, AAEL009760, AAEL006854, AAEL004120 and AAEL007591, are downregulated [8]. The expressions of AAEL001634 and AAEL007591 are turned off post CHIKV infection (Table S5A,B). The order of gene expression of rest of the genes along with the fold change is AAEL009760 (1.4×) > AAEL012064 (1.3×) > AAEL006854 (1.1×) > AAEL004120 (1.0×). AAEL001650 (12.3×) is the only upregulated gene (Table S5A,B).

In the case of DENV2 at 14-dpi, eight genes are downregulated [8]. AAEL007591, AAEL009553, AAEL015136 and AAEL015137 are turned off in DENV2-infected salivary gland (Table S5C,D). The order of gene expression of other genes along with the fold change is AAEL001634 (3.5×) > AAEL006854 (2.8×) > AAEL004120 (1.7×) > AAEL012064 (1.5×). On the other hand, three genes are upregulated: AAEL009556 (6.4×) > AAEL001650 (5.1×) > AAEL009555 (1.3×) (Table S5C,D).

In ZIKV-infected salivary gland at 14-dpi, seven genes are downregulated [8]. AAEL009553, AAEL009555, AAEL009556 and AAEL015137 are completely turned off post ZIKV infection (Table S5E,F). AAEL009760 (2.6×) > AAEL012064 (1.6×) > AAEL001634 (1.4×) is the order of gene expression for the other downregulated genes. On the other hand, three genes are upregulated in the following order: AAEL007591 (2.6×) > AAEL006854 (1.5×) > AAEL001650 (1.3×). AAEL007592 is expressed only post ZIKV infection (Table S5E,F).

In summary, seven genes (AEL001654, AAEL009956, AAEL015139, AAEL015140, AAEL020314, AAEL026174 and AAEL025109) are not expressed in the uninfected or infected (CHIKV, DENV2 or ZIKV) salivary gland. Interestingly, in the salivary gland infected with CHIKV, DENV2 or ZIKV, AAEL012064 and AAEL001634 are downregulated and AAEL01650 is upregulated. As these differential expression profiles are derived from the same strain of mosquitos (Singapore), we speculate that these three genes are important in replication of arboviruses in the salivary gland. On the other hand, AAEL006854 and AAEL007591 are downregulated in the salivary gland infected with CHIKV and DENV2 but are upregulated when infected with ZIKV. The contrasting expression profiles may be due to different roles played by AAEL006854 and AAEL007591 in the infection process of CHIKV, DENV2 or ZIKV. Similar to the midgut, most genes were downregulated rather than upregulated in the infected salivary gland. AAEL012064 is the only gene downregulated in both midgut and salivary gland tissues in all virus-infected mosquitos.

Taken together, virus infections appear to affect the expression of several NPC2 genes, indicating their potential role in arboviral transmission. Further experiments, including the knockdown and knockout of specific NPC2 genes [24], are needed to fully understand their roles and their impact on viral transmissions.

## 3. Discussion

In mammals, only one highly conserved, ubiquitously expressed gene encodes for NPC2 protein, which participates in cholesterol binding and transport in all cells [19]. By contrast, arthropods express several NPC2 proteins, whose functions are unclear. For example, *Drosophila melanogaster* has eight NPC2 proteins. However, the function of only one protein (NPC2a) is well characterised [12]. It plays a vital role in cholesterol transport, ecdysteroid biosynthesis and moulting [12]. In *A. aegypti*, the NPC2 superfamily contains 20 proteins that are diverse and expressed in the midgut, salivary gland, brain, antenna, and proboscis [21]. These NPC2 proteins are poorly characterised and very little information on their function and structure–function relationships are available. A comparison of *A. aegypti* NPC2 proteins with bovine NPC2 protein and its residues involved in binding cholesterol helped us understand the potential interaction of mosquito NPC2 proteins with cholesterol or related sterols (Figure 3C). Several *A. aegypti* NPC2 proteins have unfavourable mutations that would disrupt the ligand binding or potentially modify the conformation of the protein, resulting in the loss of ligand binding (Figure 3C). Based on our analysis, we identified four NPC2 proteins (AAEL006854, AAEL020314, AAEL009553 and AAEL009556) as potential cholesterol-binding proteins (Figure 3C). The structures of these four mosquito NPC2 proteins predicted via molecular modelling were compared with apo and cholesterol bound bovine NPC2. The ligand-binding residues are positioned in a similar manner, creating a cholesterol-binding pocket, and will allow sterol to fit and bind as in bovine NPC2.

Most residues of mosquito NPC2 interacting with human NPC1 for cholesterol transfer are altered in all four potential cholesterol-binding proteins (Figure 3D). Similarly, *A. aegypti* NPC1 also have non-conserved mutations replacing the residues of the human NPC1 functional site (Figure S4). Hence, it is unclear whether NPC1 and NPC2 proteins in the mosquito are involved in cholesterol transfer or not. However, their roles could be different from those of vertebrate NPC2 protein. Since none of the *A. aegypti* NPC2 genes appear to be ubiquitously expressed, it would be interesting to understand the players and the mechanism of intracellular cholesterol transport in invertebrates. Interestingly, Jupatanakul et al. showed that AAEL006854 (a NPC2) and AAEL009531 (a NPC1) act as agonists of dengue serotype 1 infection in the midgut [20]. The role of these proteins in cholesterol transport has not been documented.

Based on the sequence identity with *Drosophila* NPC2a gene, we propose that AAEL006854 and/or AAEL020314 may be involved in ecdysteroid biosynthesis and moulting [12]. Therefore, these NPC2 genes are potential targets for knockout or conditional knockdown to produce transgenic mosquitos with inefficient moulting, which, in turn, could lead to mosquito eradication.

As discussed before, the potential cholesterol-binding NPC2 genes have a range of expression in adult female *A. aegypti* tissues. The varied expression of these genes in different mosquito tissues may indicate tissue-specific roles played by them. The interaction of these NPC2 proteins with various sterols as well as with *A. aegypti* NPC1 proteins may help delineate their physiological functions. On the other hand, the fatty-acid-binding gene, AAEL025109, is not expressed in adult female *A. aegypti* in detectable levels. Expression studies in male tissues or different developmental stage may help understand the expression and semiochemical binding role of this gene.

Studies suggest that the NPC2 protein family in arthropods is involved in chemical communication by binding to certain semiochemicals [22,23]. The NPC2 of the worker ant *Camponotus japonicus* that binds to oleic acid is proposed to aid in chemical communication [21]. We compared mosquito NPC2 proteins to the residues of the binding pocket of ant NPC2 (Figure 6C). Most of the mosquito proteins presented an unfavourable environment for fatty acid binding. Only AAEL025109 comprised conserved residues for fatty acid binding (Figure 6C). The modelled structure of AAEL025109 shows a fair similarity to apo and oleic acid bound ant NPC2. However, the two lysines are not in the proximity to form strong bonds with oleic acid. Thus, we predicted that this protein may bind to long-chain fatty acid or alcohol in a different orientation.

Unlike ant NPC2, AAEL025109 is not expressed in the antenna and, thus, may have a distinct physiological role, whereas the highly expressed genes in the antenna (AAEL006854, AAEL020314, AAEL004120 and AAEL012064) cannot bind to a long-chain fatty acid due to various mutations in the binding residues, especially the mutation of the double lysine site that forms the hydrogen bonds with the fatty acid (Figure 6C). Further, it is not clear whether AAEL025109 is expressed in the antenna or other tissues of male mosquitos or in the egg, larval or pupal stages. Tissue-specific expression and interaction with ligands may help decipher AAEL025109′s physiological role.

NPC2 genes are differentially expressed in both the midgut and the salivary gland during CHIV, DENV2 and ZIKV arbovirus infections. The varied expression of the 20 mosquito NPC2 genes during different infections may suggest that each of these genes has some role in arboviral infections. Future studies can focus on studying gene mutants for understanding this salivary protein’s role in these infections.

## 4. Materials and Methods

### 4.1. Analyses of Nucleotide and Protein Sequences of NPC2 Genes

The DNA, mRNA and protein sequences for 20 *A. aegypti* NPC2 genes were downloaded from VectorBase [25]. The protein sequences were analysed for signal peptides using SignalP 5.0 [26]. The nucleotide and protein sequences were aligned using the Clustal Omega bioinformatics tool [27]. The similarities between mRNAs and proteins, individually, were visualised as heatmaps using Matplotlib [28]. The genome-wide distribution of NPC2 genes on the three chromosomes of *A. aegypti* was analysed to understand the location of genes and identify the gene clusters in each chromosome. The relationship between the distance among the genes within the cluster and their sequence similarities were analysed. Intron–exon structures, chromosome location, and the gene and protein sizes of all NPC2 genes were studied. We also examined the synteny of two genes sharing the highest identity.

The evolutionary history was inferred by using the Maximum Likelihood method and the Whelan and Goldman model for the mature protein sequences [29]. The bootstrap consensus tree inferred from 1000 replicates [30] is taken to represent the evolutionary history of the taxa analysed [30]. Branches corresponding to partitions reproduced in less than 50% bootstrap replicates are collapsed. The percentage of replicate trees in which the associated taxa clustered together in the bootstrap test (1000 replicates) are shown next to the branches [30]. Initial tree(s) for the heuristic search were obtained automatically by applying the Neighbour-Joining and BioNJ algorithms to a matrix of pairwise distances estimated using the JTT model, and then selecting the topology with superior log likelihood value. A discrete Gamma distribution was used to model the evolutionary rate differences among the sites (5 categories (+*G*, parameter = 7.8290)). The rate variation model allowed for some sites to be evolutionarily invariable ([+*I*], 3.95% sites). This analysis involved 24 amino acid sequences. There was a total of 177 positions in the final dataset. The tree was rooted using the human NPC2 (NP_006423.1). All evolutionary analyses, which included sequence alignment with MUSCLE, model selection and tree generation, were conducted in MEGA X [31,32].

### 4.2. Structure–Function Relationships of NPC2 Proteins

To identify the residues in *A. aegypti* NPC2 proteins involved in the interaction with cholesterol or fatty acid, we compared their sequences with the amino acid residues that are involved in binding to cholesterol or fatty acid in the crystal structures of reference proteins, human (PDB 5KWY), bovine (PDB 2HKA), and ant (PDB 3WEB) NPC2 proteins complexed with cholesterol or fatty acid [16,20,22]. The structures were visualised and analysed using PyMOL 2.5 [19]. The residues of our proteins were compared to the binding residues of reference proteins using heatmaps created with Matplotlib [28]. The identical residues were marked in green, conserved residues in yellow, and non-conserved residues in red. This provided a simple visual scheme to identify NPC2 proteins that would potentially bind sterol or fatty acid.

### 4.3. Molecular Modelling of NPC2 Proteins

Using AlphaFold2 [33,34], we predicted the structures of the potential cholesterol-binding proteins AAEL006854, AAEL020314, AAEL009553 and AAEL009556, and fatty-acid-binding protein AAEL025109. The predicted Local Distance Difference Test (pLDDT), per-residue estimate of the structure’s confidence on a scale from 0 to 100, was produced for each model of each protein. The Predicted Aligned Error (PAE) graphs for each model of each protein were noted. The top rank, low PAE, high pLDDT model was concluded to be the best model for AAEL006854, AAEL020314, AAEL009553, AAEL009556 and AAEL025109. These predicted structures were used for further analysis.

### 4.4. Comparison and Superimposition of NPC2 Proteins

Each of the predicted cholesterol-binding protein structure was compared with the apo and cholesterol sulphate-bound bovine NPC2 (PDB 1NEP and 2HKA, respectively). Similarly, the predicted fatty-acid-binding protein structure was compared with apo and oleic-acid-bound ant NCP2 (PDB 3WEA and 3WEB, respectively). All structures were superimposed individually and together onto bovine NPC2 and ant NPC2, and analysed using PyMOL [19]. The RMSD values of all heavy atoms for each structure were recorded.

### 4.5. Expression of NPC2 Genes in Mosquito Tissues

The expression profiles of NPC2 genes were evaluated using reads from transcriptomic studies [5,6,8,9,24,35]. The raw reads of female *A. aegypti* are available for the midgut, salivary gland, antenna, brain, proboscis and whole body [5,6,8,9,24,35]. The mosquitos were blood-fed in the transcriptome studies of the midgut, salivary gland and whole body, whereas non-blood fed mosquitos were used for the transcripts from antenna, brain and proboscis. We aligned and quantified the raw reads of RNAseq data from the midgut, antenna, brain, proboscis, and whole body using Bowtie2 [36] and RSEM [37] to evaluate the expression of NPC2 genes. For the salivary gland, the reads were normalised using DESeq2 [8].

The raw reads of transcripts from only the midgut and salivary gland of infected female *A. aegypti* are available [5,6,8,9,24]. The mosquitos were infected orally with CHIKV, DENV1 or DENV2 in the midgut and dissected at 1-dpi, 4-dpi and 14-dpi for CHIKV, DENV1, or DENV2, respectively [6,8,24]. In contrast, the salivary gland was infected orally with CHIKV, DENV2 or ZIKV and dissected at 7-dpi for CHIKV and 14-dpi for DENV2 or ZIKV [9]. Genes that showed a differential expression of over 20% of the control in the midgut and salivary gland were analysed. Analyses of the differential expression of NPC2 genes allowed us to effectively compare their expression pattern in midgut and salivary gland tissues.

## 5. Conclusions

Unlike the single conserved, ubiquitously expressed NPC2 protein involved in cholesterol trafficking vertebrates, *A. aegypti* has 20 diverse NPC2 proteins. By comparing their sequences and the modelled structures to human and bovine NPC2 proteins, we identified four potential sterol-binding proteins in *A. aegypti*. Two of them may play a key role in ecdysteroid biosynthesis and moulting. We also identified one *A. aegypti* NPC2 protein as potential fatty-acid-binding protein by comparing it to ant NPC2 protein. Further, we provided insights on the differential expression of NPC2 genes in CHIKV, DENV2 and ZIKV arboviral infections.

## Data Availability

Data are contained within the article or in the Supplementary Materials.

## Supplementary Materials

The following supporting information can be downloaded at: https://www.mdpi.com/article/10.3390/ijms25031684/s1. References [5,6,8,9,24,35] are cited in Supplementary Materials.
