# Supplementary material for: Niemann–Pick Type C2 Proteins in Aedes aegypti: Molecular Modelling and Prediction of Their Structure–Function Relationships
Source: Int J Mol Sci. 2024 Jan 30;25(3):1684. doi: 10.3390/ijms25031684 (PMC10855982; doi:10.3390/ijms25031684)
Supplement: Supplementary file 1 [file ijms-25-01684-s001.zip › ijms-2766884-supplementary.pdf]

Supplementary Information
to
Niemann–Pick Type C2 Proteins in *Aedes aegypti*: Molecular Modelling and Prediction of Their Structure–Function Relationships

Prathigna Jaishankar Thambi [1], Cassandra M. Modahl [2,3,4,*] and R. Manjunatha Kini [2,5,6,*]

1 King's College London, London SE1 1UL, UK; prathignathambi@gmail.com
2 Department of Biological Sciences, Faculty of Science, National University of Singapore, Singapore 117543, Singapore
3 Centre for Snakebite Research and Interventions, Liverpool School of Tropical Medicine, Liverpool L3 5QA, UK
4 Vector Biology Department, Liverpool School of Tropical Medicine, Liverpool L3 5QA, UK
5 Department of Pharmacology, Yong Loo Lin School of Medicine, National University of Singapore, Singapore 117600, Singapore
6 Department of Biochemistry and Molecular Biology, VCU School of Medicine, Virginia Commonwealth University, Richmond, VA 23298, USA
* Correspondence: cassandra.modahl@lstmed.ac.uk (C.M.M.); dbskinim@nus.edu.sg (R.M.K.)

Supplementary Figures

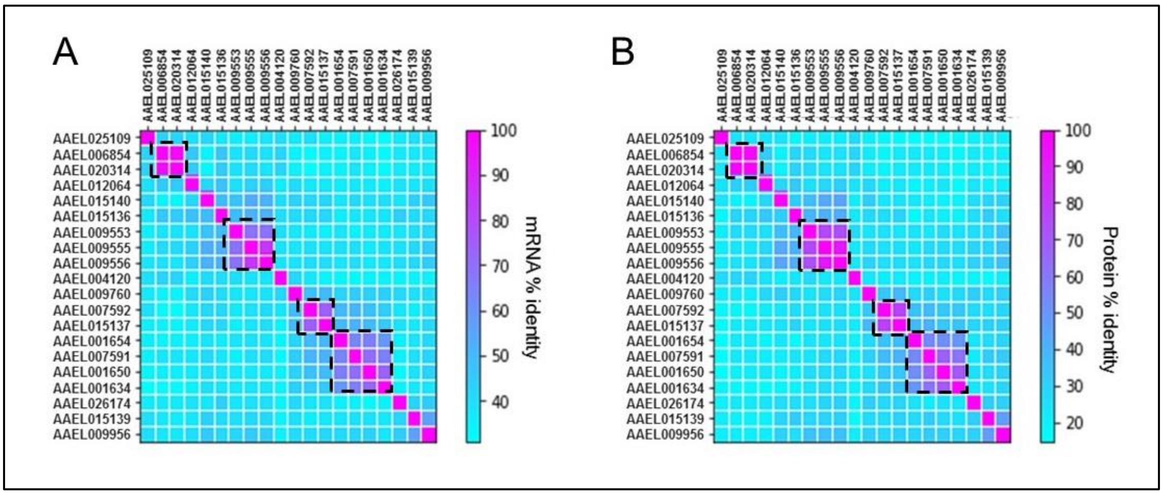

Figure S1. Sequence identity of *A. aegypti* NPC2 mRNA and mature proteins. The heatmaps depict the mRNA (**A**) and protein (**B**) identity (%) of 20 *A. aegypti* NPC2. Four set of genes - (AAEL006854 and AAEL020314), (AAEL009553, AAEL009555 and AAEL009556), (AAEL001654, AAEL007591, AAEL001650, and AAEL001634) and (AAEL007592 and AAEL015137) form clusters of high identity on the heatmap and are marked with black dotted boxes.

	AAEL007592	AAEL007591	AAEL015137	AAEL015139	AAEL009956	AAEL026174	AAEL015136	AAEL015140	AAEL025109
AAEL007592		37.33	77.63	25.5	29.05	28.08	26.53	21.48	15.15
AAEL007591	51.43		41.06	30.41	32.65	21.92	24.49	22.97	13.64
AAEL015137	73.64	52.85		29.14	30.67	29.25	29.05	23.84	15.04
AAEL015139	39.96	42.67	40.96		47.33	30.61	30.61	28.00	21.97
AAEL009956	41.03	44.7	44.25	56.07		30.14	25.85	30.00	18.18
AAEL026174	36.05	34.85	36.91	38.02	41.69		26.21	26.71	18.37
AAEL015136	40.33	38.21	41.22	41.78	45.11	45.66		31.76	17.56
AAEL015140	40.32	39.22	40.67	38.48	41.47	39.69	49.66		23.13
AAEL025109	39.02	35.51	38.05	40.23	39.76	37.03	35.32	40.55	

Figure S2. Sequence identity among nine NPC2 genes forming a large cluster in chromosome 1. The lowest percentage identity in mRNA (Red) is about 35%, whereas it is about 14% in proteins (Blue). NPC2 expressing low identity are marked in green boxes, whereas the high percentages are marked in purple boxes. Despite their presence in a cluster, only two NPC2, AAEL007592 and AAEL015137, show >73.5% identity, while all others show poor similarity.

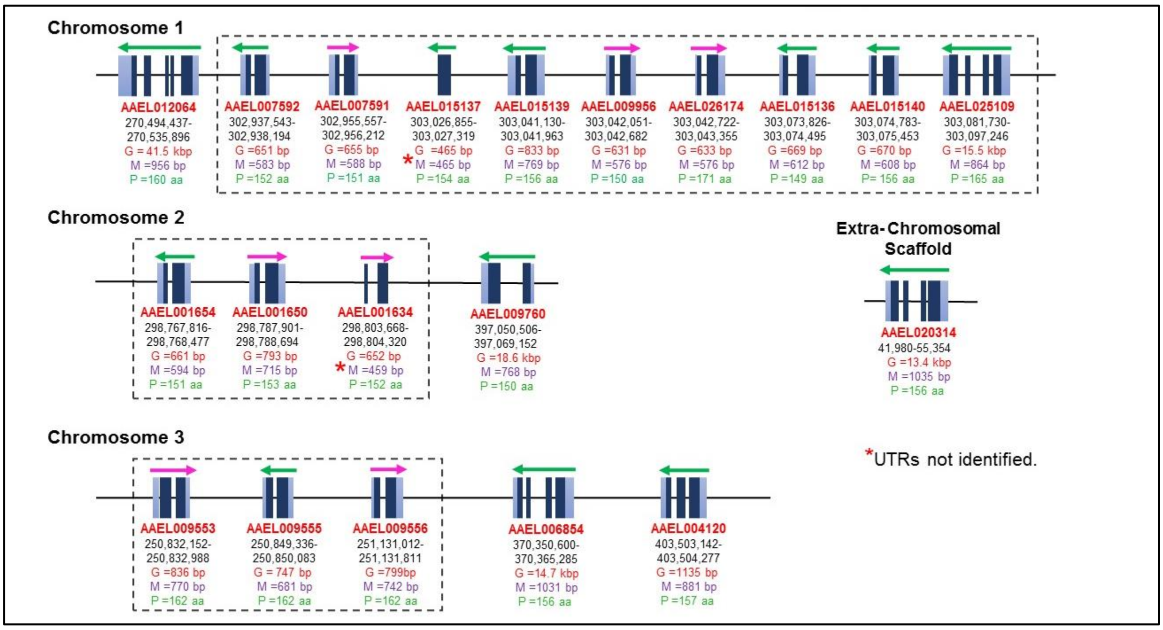

Figure S3. Intron-exon structure, orientation, location and sizes of gene, mRNA and full-length protein of *A. aegypti* NPC2 genes. Intron-exon profiles for each gene is indicated with blue boxes, exons (dark blue boxes), introns (gaps between boxes), and 5' and 3' UTRs (light blue boxes). The UTRs of some genes (*) have not been identified. The chromosome location, and sizes of gene (G), mRNA (M) and protein (P) are indicated in appropriate units of base pairs and amino acid residues. The direction of transcription is shown above each gene.

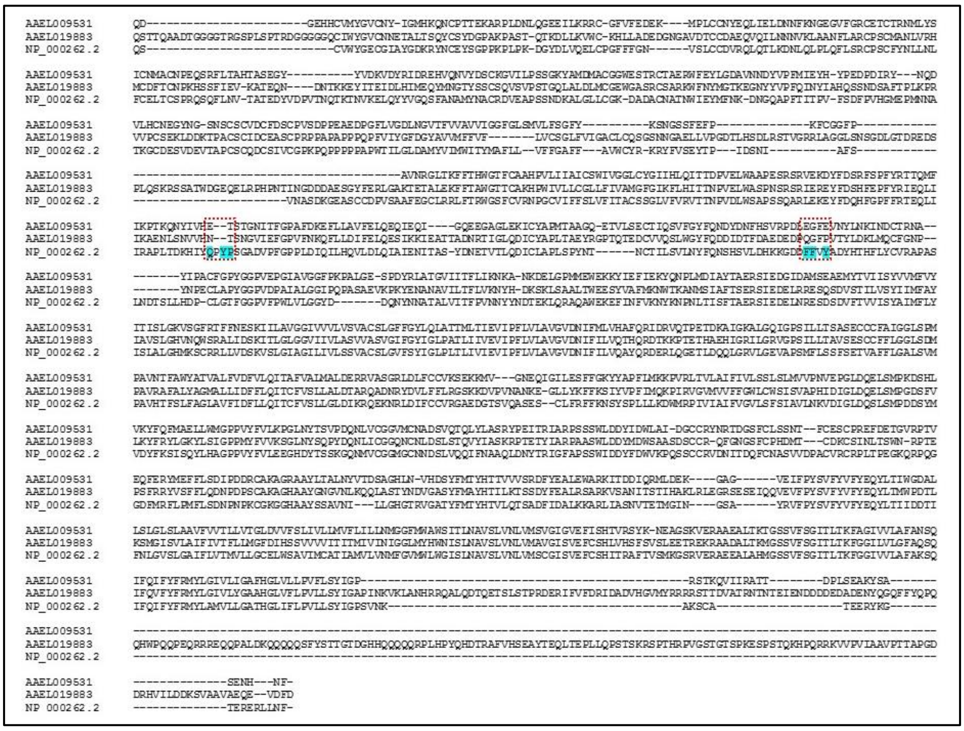

Figure S4. Sequence alignment of mature *A. aegypti* and *Homo sapiens* NPC1 proteins. The sequences of mature NPC1 proteins of *A. aegypti* (AAEL009531 and AAEL019883) and *Homo sapiens* (NP_000262.2) were aligned using Clustal Omega. The six residues of human NPC1 that interact with NPC2 while participating in cholesterol transfer are marked in teal. All of these residues are mutated in *A. aegypti* NPC1 (red dotted boxes).

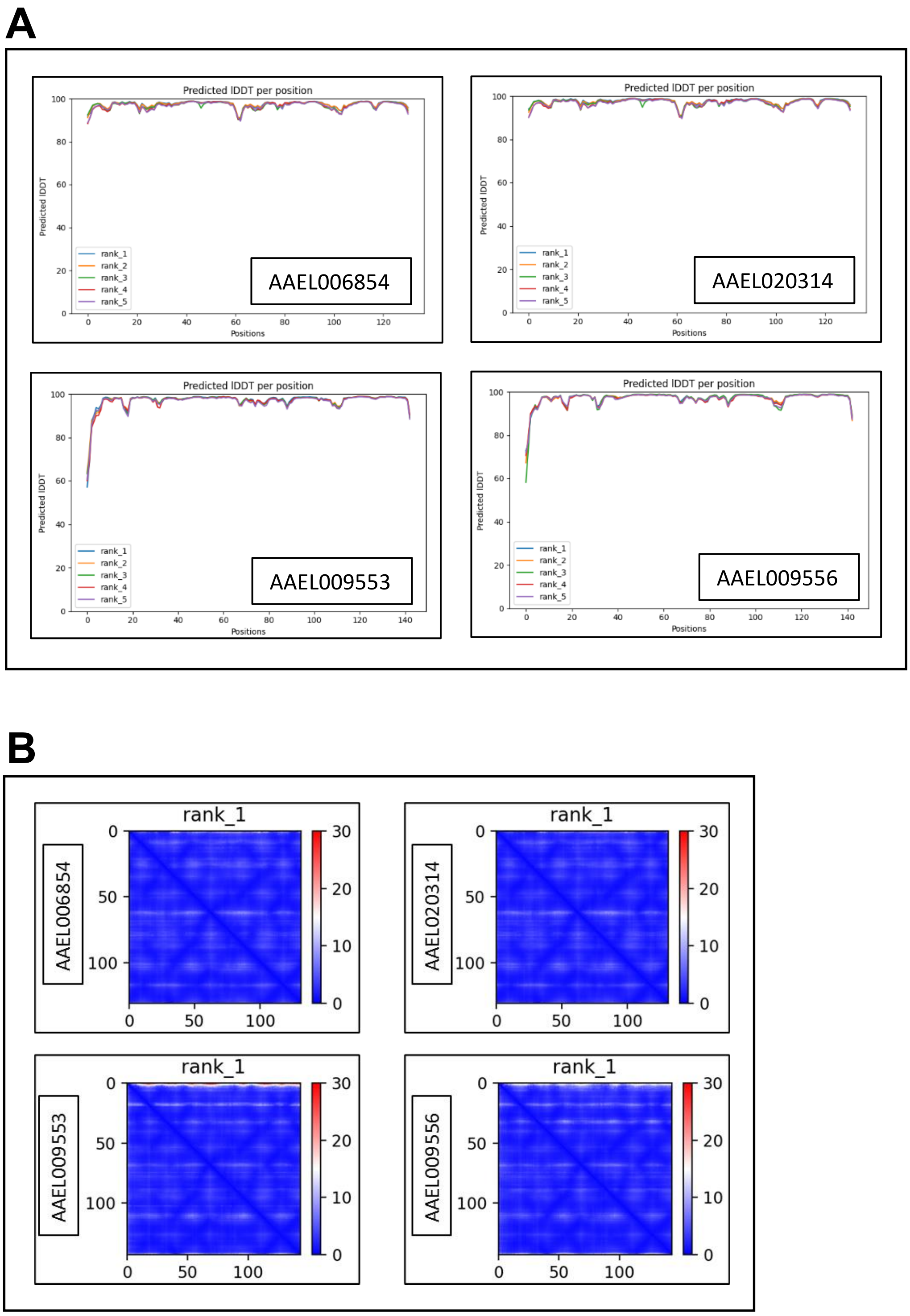

Figure S5. pLDDT and PAE graphs predicted cholesterol binding NPC2 proteins. The structures of four potential cholesterol binding *A. aegypti* NPC2 proteins was predicted using AlphaFold2. The top rank, low PAE and high pLDDT structures were concluded as the best models for each protein.

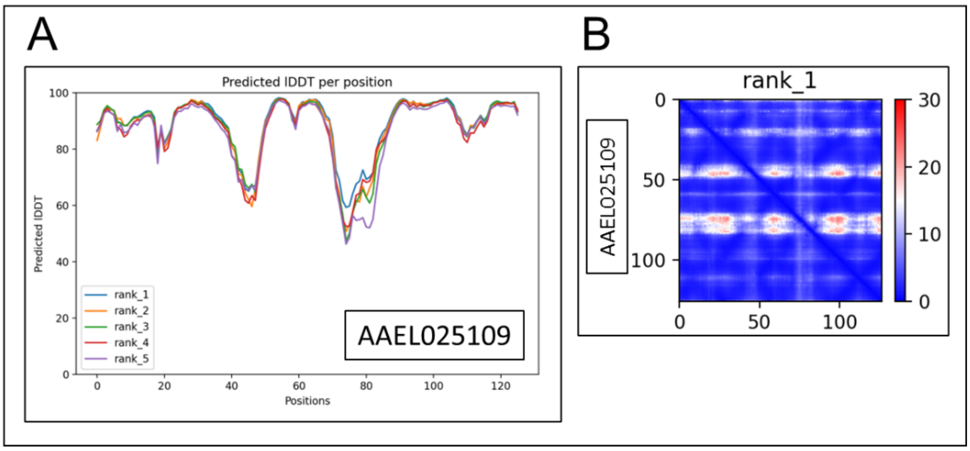

Figure S6. pLDDT and PAE graphs predicted fatty acid binding NPC2 protein. The structure of a potential fatty acid binding *A. aegypti* NPC2 protein, AAEL025109, was predicted using AlphaFold2. The top rank, low PAE and high pLDDT structure was concluded as the best model.

Supplementary Tables

Table S1. RMSD (all heavy atoms) of four potential cholesterol binding NPC2 protein structures with Apo (1NEP) and cholesterol-bound (2HKA) bovine NPC2.

	Apo-NPC2 (Bovine, 1NEP) (RMSD, Å)	NPC2-cholesterol complex (Bovine, 2HKA) (RMSD, Å)
AAEL006854	1.168	0.746
AAEL020314	1.138	0.751
AAEL009553	1.123	1.184
AAEL009556	1.181	1.075

Table S2. RMSD (all heavy atoms) of one potential fatty acid binding NPC2 protein structure with Apo (3WEA) and oleic acid-bound (3WEB) ant NPC2.

	Apo-NPC2 (Ant, 3WEA) (RMSD, Å)	NPC2-fatty acid complex (Ant, 3WEB) (RMSD, Å)
AAEL025109	2.097	2.420

Table S3. Expression of NPC2 genes in tissues of *A. aegypti*

(A) Expression of NPC2 genes in antenna, brain, proboscis and whole body

	Antenna*	Brain*	Proboscis*	Whole Body#
AAEL001634	0.00	0.00	0.00	0.00
AAEL001650	0.00	6724.62	0.00	0.00
AAEL001654	0.00	325.62	0.00	0.00
AAEL004120	97325.56	255785.10	10707.65	108777.13
AAEL006854	1747848.90	17104.74	171139.30	116420.10
AAEL007591	0.00	0.00	0.00	39934.75
AAEL007592	825.46	0.00	0.00	27754.65
AAEL009553	380.90	0.00	0.00	0.00
AAEL009555	0.00	0.00	0.00	0.00
AAEL009556	0.00	397.33	0.00	14802.48
AAEL009760	419.75	0.00	0.00	1257004.01
AAEL009956	0.00	0.00	0.00	0.00
AAEL012064	53774.53	2060221.71	2351248.48	416319.74
AAEL015136	0.00	0.00	0.00	192459.25
AAEL015137	0.00	0.00	0.00	0.00
AAEL015139	0.00	0.00	0.00	0.00
AAEL015140	0.00	0.00	0.00	0.00
AAEL020314	685575.39	5704.24	74437.94	87884.96
AAEL026174	0.00	0.00	0.00	1745.58
AAEL025109	0.00	0.00	0.00	0.00

*Each value is the average of individual sets of 100-220 antenna, 9-18 brain and 275-797 proboscis of Liverpool mosquito strain [1].
#Each value is the average 3 individual whole-body of Liverpool mosquito strain [2].

(B) Expression of NPC2 genes in midgut

	Day 1*	Day 4#	Day 14§
AAEL001634	0.00	0.00	0.00
AAEL001650	365.74	1184.16	194.51
AAEL001654	280.09	320.44	0.00
AAEL004120	336.87	195.74	659.59
AAEL006854	88.65	717.35	716.79
AAEL007591	198676.21	252027.37	712972.49
AAEL007592	26885.00	3021.88	87141.99
AAEL009553	0.00	0.00	0.00
AAEL009555	0.00	0.00	183.64
AAEL009556	162543.71	12447.31	17460.29
AAEL009760	311665.17	166901.01	664926.92
AAEL009956	1887.16	161.41	844.64
AAEL012064	852.68	4131.45	2686.99
AAEL015136	2790862.96	1997498.04	888536.30
AAEL015137	2352.97	5564.54	9351.43
AAEL015139	1152.91	0.00	1142.74
AAEL015140	886.85	1776.92	190.45
AAEL020314	177.40	4304.39	473.57
AAEL026174	49034.14	35249.04	21863.03
AAEL025109	0.00	0.00	0.00

*Each value is the average of 15 individual midguts of Puerto Rico mosquito strain [3].
#Each value is the average of 45 individual midguts of Thailand mosquito strain [4].
§Each value is the average of 10 individual midguts of Mexico mosquito strain [5].

(C) Expression of NPC2 genes in salivary glands

	Day 7*	Day 14*§	Day 14*§
AAEL001634	1.04	1.69	1.91
AAEL001650	0.45	0.93	1.05
AAEL001654	0.00	0.00	0.00
AAEL004120	277.74	323.26	364.30
AAEL006854	7.29	14.01	15.79
AAEL007591	0.45	0.55	0.62
AAEL007592	0.00	0.00	0.00
AAEL009553	0.00	0.76	0.86
AAEL009555	0.00	0.38	0.43
AAEL009556	0.45	0.38	0.43
AAEL009760	1.94	5.75	6.49
AAEL009956	0.00	0.00	0.00
AAEL012064	112.44	186.06	209.75
AAEL015136	0.35	0.93	1.05
AAEL015137	0.00	0.55	0.62
AAEL015139	0.00	0.00	0.00
AAEL015140	0.00	0.00	0.00
AAEL020314	0.00	0.00	0.00
AAEL026174	0.00	0.00	0.00
AAEL025109	0.00	0.00	0.00

*Each value is the average of two independent sets of 20 pairs of salivary glands [6].
§Performed on two independent days [6].

Table S4. Differential expression in the midgut infected with CHIKV, DENV1 and DENV2

(A) Differential expression of genes in the midgut infected with CHIKV

	Control MG Day 1 *	CHIKV MG 1-dpi*
AAEL001634	0.00	0.00
AAEL001650	365.74	0.00
AAEL001654	280.09	0.00
AAEL004120	336.87	196.51
AAEL006854	88.65	0.00
AAEL007591	198676.21	304251.16
AAEL007592	26885.00	30985.36
AAEL009553	0.00	0.00
AAEL009555	0.00	111.09
AAEL009556	162543.71	114643.93
AAEL009760	311665.17	427435.53
AAEL009956	1887.16	2010.28
AAEL012064	852.68	453.10
AAEL015136	2790862.96	2695051.43
AAEL015137	2352.97	3611.56
AAEL015139	1152.91	1061.27
AAEL015140	886.85	825.43
AAEL020314	177.40	353.76
AAEL026174	49034.14	55193.21
AAEL025109	0.00	0.00

*Each value is the average of 15 individual midguts of Puerto Rico mosquito strain [3].

(B) Fold change of genes that demonstrate high differential expression in the midgut post-CHIKV infection

CHIKV	Down (D)
AAEL001650	D to zero
AAEL001654	D to zero
AAEL006854	D to zero
AAEL012064	1.9x
AAEL004120	1.7x
	Up (U)
AAEL009555	U from zero
AAEL020314	2.0x
AAEL007591	1.5x
AAEL015137	1.5x
AAEL009760	1.4x

D, shut down to Zero expression.
U, initiation of expression from Zero.

(C) Differential expression of genes in the midgut infected with DENV1

	Control MG Day 4 #	DENV1 MG 4-dpi#
AAEL001634	0.00	0.00
AAEL001650	1184.16	0.00
AAEL001654	320.44	0.00
AAEL004120	195.74	18.35
AAEL006854	717.35	50.13
AAEL007591	252027.37	3303.94
AAEL007592	3021.88	1751.66
AAEL009553	0.00	0.00
AAEL009555	0.00	0.00
AAEL009556	12447.31	555.57
AAEL009760	166901.01	2432057.66
AAEL009956	161.41	0.00
AAEL012064	4131.45	1020.92
AAEL015136	1997498.04	36507.28
AAEL015137	5564.54	73.46
AAEL015139	0.00	0.00
AAEL015140	1776.92	72.41
AAEL020314	4304.39	927.46
AAEL026174	35249.04	3925.39
AAEL025109	0.00	0.00

#Each value is the average of 45 individual midguts of Thailand mosquito strain [4].

(D) Fold change of genes that demonstrate high differential expression in the midgut post-DENV1 infection

DENV1	Down (D)
AAEL001650	D to zero
AAEL001654	D to zero
AAEL009956	D to zero
AAEL007591	76.3x
AAEL015137	75.7x
AAEL015136	54.7x
AAEL015140	24.5x
AAEL009556	22.4x
AAEL006854	14.3x
AAEL004120	10.7x
AAEL026174	9.0x
AAEL020314	4.6x
AAEL012064	4.0x
AAEL007592	1.7x
	Up
AAEL009760	14.6x

D, shut down to Zero expression.
U, initiation of expression from Zero.

(E) Differential expression of genes in the midgut infected with DENV2

	Control MG Day 14§	DENV2 MG 14-dpi§
AAEL001634	0.00	0.00
AAEL001650	194.51	340.78
AAEL001654	0.00	0.00
AAEL004120	659.59	342.40
AAEL006854	716.79	1171.94
AAEL007591	712972.49	623372.81
AAEL007592	87141.99	61405.28
AAEL009553	0.00	0.00
AAEL009555	183.64	0.00
AAEL009556	17460.29	14094.82
AAEL009760	664926.92	822380.22
AAEL009956	844.64	696.38
AAEL012064	2686.99	1113.14
AAEL015136	888536.30	856842.53
AAEL015137	9351.43	10471.88
AAEL015139	1142.74	1048.72
AAEL015140	190.45	524.32
AAEL020314	473.57	67.46
AAEL026174	21863.03	17614.21
AAEL025109	0.00	0.00

§Each value is the average of 10 individual midguts of Mexico mosquito strain [5].

(F) Fold change of genes that demonstrate high differential expression in the midgut post-DENV2 infection

DENV2	Down (D)
AAEL009555	D to zero
AAEL020314	7.0x
AAEL012064	2.4x
AAEL004120	1.9x
AAEL007592	1.4x
	Up
AAEL015140	2.8x
AAEL001650	1.8x
AAEL006854	1.6x
AAEL009760	1.2x

D, shut down to Zero expression.
U, initiation of expression from Zero.

Table S5. Differential expression in salivary glands infected with CHIKV, DENV2 and ZIKV

(A) Differential expression of genes in the salivary gland infected with CHIKV

	Control SG Day 7*	CHIKV SG 7-dpi*
AAEL001634	1.04	0.00
AAEL001650	0.45	5.54
AAEL001654	0.00	0.00
AAEL004120	277.74	272.52
AAEL006854	7.29	6.69
AAEL007591	0.45	0.00
AAEL007592	0.00	0.00
AAEL009553	0.00	0.00
AAEL009555	0.00	0.00
AAEL009556	0.45	0.00
AAEL009760	1.94	1.41
AAEL009956	0.00	0.00
AAEL012064	112.44	84.78
AAEL015136	0.35	0.00
AAEL015137	0.00	0.00
AAEL015139	0.00	0.00
AAEL015140	0.00	0.00

* Each value is the average of two independent sets of 20 pairs of salivary glands [6].
§Performed on two independent days [6].

(B) Fold change of genes that demonstrate high differential expression in the salivary gland post-CHIKV infection

CHIKV	Down (D)
AAEL001634	D to zero
AAEL007591	D to zero
AAEL009760	1.4x
AAEL012064	1.3x
AAEL006854	1.1x
AAEL004120	1.0x
AAEL001650	12.3x

D, shut down to Zero expression.
U, initiation of expression from Zero.

(C) Differential expression of genes in the salivary gland infected with DENV2

	Control SG Day 14*	DENV2 SG 14-dpi *
AAEL001634	1.69	0.48
AAEL001650	0.93	4.72
AAEL001654	0.00	0.00
AAEL004120	323.26	195.34
AAEL006854	14.01	5.09
AAEL007591	0.55	0.00
AAEL007592	0.00	0.00
AAEL009553	0.76	0.00
AAEL009555	0.38	0.48
AAEL009556	0.38	2.43
AAEL009760	5.75	5.69
AAEL009956	0.00	0.00
AAEL012064	186.06	126.29
AAEL015136	0.93	0.00
AAEL015137	0.55	0.00
AAEL015139	0.00	0.00
AAEL015140	0.00	0.00

* Each value is the average of two independent sets of 20 pairs of salivary glands [6].
§Performed on two independent days [6].

(D) Fold change of genes that demonstrate high differential expression in the salivary gland post-DENV2 infection

DENV2	Down (D)
AAEL007591	D to zero
AAEL009553	D to zero
AAEL015136	D to zero
AAEL015137	D to zero
AAEL001634	3.5x
AAEL006854	2.8x
AAEL004120	1.7x
AAEL012064	1.5x
	Up
AAEL009556	6.4x
AAEL001650	5.1x
AAEL009555	1.3x

D, shut down to Zero expression.
U, initiation of expression from Zero.

(E) Differential expression of genes in the salivary gland infected with ZIKV

	Control SG Day 14*	ZIKV SG 14-dpi*
AAEL001634	1.91	1.40
AAEL001650	1.05	1.40
AAEL001654	0.00	0.00
AAEL004120	364.30	394.85
AAEL006854	15.79	24.11
AAEL007591	0.62	1.61
AAEL007592	0.00	4.29
AAEL009553	0.86	0.00
AAEL009555	0.43	0.00
AAEL009556	0.43	0.00
AAEL009760	6.49	2.47
AAEL009956	0.00	0.00
AAEL012064	209.75	129.77
AAEL015136	1.05	1.07
AAEL015137	0.62	0.00
AAEL015139	0.00	0.00
AAEL015140	0.00	0.00

* Each value is the average of two independent sets of 20 pairs of salivary glands [6].
§Performed on two independent days [6].

(F) Fold change of genes that demonstrate high differential expression in the salivary gland post-ZIKV infection

ZIKV	Down (D)
AAEL009553	D to zero
AAEL009555	D to zero
AAEL009556	D to zero
AAEL015137	D to zero
AAEL009760	2.6x
AAEL012064	1.6x
AAEL001634	1.4x
	Up (U)
AAEL007592	U from zero
AAEL007591	2.6x
AAEL006854	1.5x
AAEL001650	1.3x

D, shut down to Zero expression.
U, initiation of expression from Zero.

References

[1] B. J. Matthews, C. S. McBride, M. DeGennaro, O. Despo, and L. B. Vosshall, “The neurotranscriptome of the Aedes aegypti mosquito,” *BMC Genomics*, vol. 17, no. 1, Jan. 2016, doi: 10.1186/s12864-015-2239-0.

[2] M. Bonizzoni *et al.*, “RNA-seq analyses of blood-induced changes in gene expression in the mosquito vector species, Aedes aegypti,” *BMC Genomics*, vol. 12, Jan. 2011, doi: 10.1186/1471-2164-12-82.

[3] S. Dong, S. K. Behura, and A. W. E. Franz, “The midgut transcriptome of Aedes aegypti fed with saline or protein meals containing chikungunya virus reveals genes potentially involved in viral midgut escape,” *BMC Genomics*, vol. 18, no. 1, May 2017, doi: 10.1186/s12864-017-3775-6.

[4] V. Raquin *et al.*, “Individual co-variation between viral RNA load and gene expression reveals novel host factors during early dengue virus infection of the Aedes aegypti midgut,” *PLoS Neglected Tropical Diseases*, vol. 11, no. 12, Dec. 2017, doi: 10.1371/journal.pntd.0006152.

[5] M. Bonizzoni, W. A. Dunn, C. L. Campbell, K. E. Olson, O. Marinotti, and A. A. James, “Complex Modulation of the Aedes aegypti Transcriptome in Response to Dengue Virus Infection,” *PLoS ONE*, vol. 7, no. 11, Nov. 2012, doi: 10.1371/journal.pone.0050512.

[6] A. Chowdhury *et al.*, “JNK pathway restricts DENV2, ZIKV and CHIKV infection by activating complement and apoptosis in mosquito salivary glands,” *PLoS Pathogens*, vol. 16, no. 8, Aug. 2020, doi: 10.1371/journal.ppat.1008754.
